# How do professional connections and relationships impact midwives’ well-being and career sustainability? A Grounded Theory study protocol

**DOI:** 10.18332/ejm/178385

**Published:** 2024-03-04

**Authors:** Lynnelle Moran, Sara Bayes, Kim Foster

**Affiliations:** 1 School of Nursing, Midwifery and Paramedicine, Australian Catholic University, Victoria, Australia; 2 School of Nursing and Midwifery, Edith Cowen University, Western Australia, Australia; 3 South Metropolitan Health Service, Fiona Stanley Hospital, Western Australia, Australia

**Keywords:** midwifery, midwives, wellbeing, career sustainability, relationships, professional connections

## Abstract

Significant attrition and projected workforce shortages within the midwifery profession are global issues. Extensive research has identified that high levels of workplace adversity, chronic stress, and increasing rates of vicarious trauma and moral injury experienced by midwives, underpin this situation. Researchers have explored midwives’ intention to stay in the midwifery workforce and identified ways to support students’ transition to professional practice. Supportive collegial relationships have been reported to be protective for new and early career midwives' well-being and resilience. However, there is a gap in knowledge and understanding of the impact and significance of professional connections and relationships for midwives across their careers. This article describes a protocol for a study designed to explore and understand how professional connections and relationships impact midwives’ well-being and career sustainability. Glaserian Grounded Theory (GT) methodology will be used to conduct the study. Constant comparison will be used to analyze data collected from in-depth interviews with midwives at various stages in their professional careers, with the aim of understanding the significance of professional connections and relationships on their well-being and career sustainability, and in understanding the potential protections and benefits. It is anticipated that the findings and theory generated from this study will have national and international implications and provide evidence about the impacts, including benefits and any potential disadvantages, of professional relationships in sustaining midwifery careers. This will be of significant value to, as well as inform, the development of midwife retention strategies.

## INTRODUCTION

Maternity systems across the world are in crisis. In recent years, they have been reported to have workforce shortages driven in part by untenable work conditions that involve high levels of workplace stressors, increasing medicalization of birth characterized by escalating rates of obstetric intervention, and higher patient acuity than previously, necessitating complex care requirements^1-4^. Untenable working conditions also include 'toxic' workplace environments, where there is a climate of fear and blame, a lack of clinical supervision, and minimal valuing of employee health and wellbeing is commonly reported^5,6^. Persistent professional disempowerment of midwives has also taken a toll^1^. The major local and global workforce shortages pose significant risks to the well-being of birthing women and their babies^7,8^.

Maternity care workplaces are known to engender high levels of anxiety, trauma, isolation, and burnout in midwives^9-13^. In relation to burnout, an international review (2020) conducted by Sidhu and the team, identified 26 organizational and personal factors that are significantly associated with the condition of burnout, and found that it was most prevalent among Western Canadian, Senegalese, and Australian midwives. Creedy et al.^10,13^ reported that the Australian midwifery workforce was under significant strain due to high rates of burnout, with 20% of midwives experiencing severe symptoms of depression, anxiety, and stress. Fenwick et al.^5^ have also reported that midwives in Victoria, Australia, have the highest rate of burnout in the country and that hospital-based/rotational midwives are at greater risk of burnout than those in other practice settings. The association between these factors and attrition from the midwifery profession is evident, with data highlighting significant losses to the profession in Australia in recent years, particularly by early-mid career midwives^1^.

Additionally, the aging midwifery workforce is a contributing factor to the crisis, with one Australian study projecting that the number of experienced midwives would significantly decline between 2018 and 2023, due to retirement^14^. A UK study conducted by Couper et al.^15^ identified that COVID-19 has amplified pre-pandemic related workforce issues and created new stressors leading to significant negative psychological impacts on midwives. Burnout experienced by midwives who worked through the peak of the pandemic, has only exacerbated losses to the profession as significant numbers of staff have either started working part-time or resigned^15,16^. In data highlighting significant losses to the profession in Australia in recent years, early-mid career midwives are the group in which attrition is highest^1^. A systematic review undertaken in 2021 identified that midwives with fewer than ten years of midwifery experience were more susceptible to burnout^11^, with early career midwives, who are commonly recognized as those qualified for five years or less, viewed as a particularly vulnerable group^17^.

Such multifactorial drivers of attrition compel a systemic shift and a solution-oriented response to develop meaningful and proactive strategies to address the issues faced by the midwifery workforce. There is an urgent need to consider effective measures for retaining those who remain in the profession. In addition, the sector needs to identify potential and existing protective factors and work, to nurture and embed these into workplaces and practices.

Research on corporate workforces outside of healthcare contexts, has consistently illuminated the broad-reaching benefits associated with positive peer relationships in the workplace, networking, and connection with one’s professional community. Geue^18^, for example, states that strong peer relationships build employee trust, respect and confidence, and work to inspire employee performance. Establishing and engaging with professional relationships has the capacity to enhance both individual and team performance while simultaneously cultivating a sense of community and belonging^19^. As might be expected, negative professional relationships can have the opposite effect. An Australian study, in which 1037 midwives were interviewed, identified that 48.6% of participants reported poor professional relationships with midwifery colleagues as a key motivator for intention to leave the profession^1^. In a midwifery context, anecdotal observations from practice suggest that strong midwifery relationships and connections help to build feelings of solidarity, strengthen one’s philosophy of practice, inspire political advocacy, promote knowledge sharing, provide opportunities for clinical reflection and mentorship, and can build confidence in both individual and collective practice.

While professional connections and relationships seem to make a difference to midwives, no research published to date specifically explores their impacts on midwives’ well-being and career sustainability. The primary aim of this research is to explore and understand how professional connections and relationships impact midwives’ well-being and career sustainability. The secondary aim is to identify and understand the potential protections and benefits of such connections and relationships. There are three specific objectives: 1) to describe the nature and significance of professional relationships and connections for midwives; 2) to understand the contextual factors that contribute to professional relationships and connections in midwifery; and 3) to describe and understand the impacts of professional relationships and connections on well-being and career sustainability for midwives at different career stages.

## METHODS

### Paradigm and methodology

Before deciding on the methodology for this study, consideration was first given on the various scientific paradigms most appropriate to situate the study. To address our aims, this study will be conducted in accordance with the *Natural Interpretivist* paradigm. In this paradigm, reality is what a person says it is. Situating the study in this paradigm enables the researchers to use a qualitative methodology to draw upon and seek understanding and meaning from participants’ lived experiences within a particular social and practice context^20^. Studies conducted within this paradigm generally employ qualitative methodologies such as Glaserian Grounded Theory (GT), which is the methodology that will be used to conduct this study.

Developed by social scientists Glaser and Strauss in 1967, Grounded Theory challenged the positivist paradigm, which was dominant in research at the time, forging a methodology that used systematic methods and robust qualitative inquiry to create theoretical explanations of human behavior^21,22^. GT uses an inductive process to generate theory, which can then be generalized and applied^23^. The Glaser and Strauss’ process, features constant comparative analysis, theoretical sampling, and theoretical coding. Emergent theories born from such robust mechanisms were considered to be ‘grounded’ as the theory emerged from the data that was simultaneously analyzed^20^. GT explains phenomena through the observation and exploration of human interactions^22^. Since the inception of Grounded Theory methodology, there have been several evolutions that have demonstrated that, as a methodology, GT is adaptable and capable of change. Glaserian GT requires that the researcher and the focus of the study remain objective. An underpinning assumption is that through analysis, categories will be derived from the data to reveal a tentative theory – the first step in generating a grounded hypothesis^22^. Due to its non-prescriptive and objective approach, this study will adopt Glaserian GT to mitigate bias and guide the development of a credible theory about the phenomenon of interest, which is the impact of professional connections and relationships on midwives’ well-being and career sustainability.

Grounded Theory is prevalent in midwifery research. For example, Bloxsome et al.^24^ undertook research using Glaserian GT to examine why midwives stay in midwifery, while Kruger and McCann^25^ used Straussian GT to generate knowledge and theory regarding midwifery scope of practice when providing care in the birth suite. Finally, Prussing et al.^26^ used Constructivist GT to examine the implementation of continuity of care models throughout regional Victoria. Each of these studies demonstrated that GT is a methodology well suited to midwifery and maternity care matters, and allows theory to be generated from the observation and interpretation of phenomena, resulting in practical recommendations. Theoretical discoveries such as those gained through GT can heighten understanding of the unique nuances that exist within the midwifery context and inform clinical behavior, policy design, and workforce planning.

### Setting

The setting for this national study comprises midwives working in public and private midwifery practice in metropolitan, rural, and community sites across Australia.

### Participants and recruitment

Participants will include Registered Midwives with a range of midwifery experience, with the intention to capture a depth of understanding of how collegial relationships impact across the continuum of professional experience. The sample will involve three groups of midwives: 1) those who have practiced midwifery for <5 years (‘early career’), those who have practiced midwifery for 5–10 years (‘mid-career’) and those who have practiced for ≥10 years. It is important to acknowledge that years of experience do not necessarily equate to expertise in midwifery practice, nor is it indicative of one’s preservation of midwifery philosophy or career fulfillment. However, it is necessary to determine parameters for inclusion, and these have been defined based on available Australian healthcare workforce attrition data and research^1,14^.

In keeping with methodological requirements, the number of participants should not be pre-determined; however, according to previous midwifery studies conducted using GT, a sample between 14–34 participants would be appropriate^4,26,27^. Three sampling techniques will be used to recruit participants and obtain data. These include purposive, snowball, and theoretical sampling. This process will commence with the recruitment of a sample by purposively selecting participants who are especially knowledgeable of the phenomenon being studied. Participants will be recruited through the Australian College of Midwives electronic bulletin and social media platforms, inviting participation in the study. This bulletin is sent to midwives across Australia. Participants will be invited to register for the study online via a protected data platform, where they will access all required participant information and complete the consent form before the interview stage. A limitation of this technique is the potential for sampling bias; however, clear integrity and ethical strategies (discussed below) will ensure data trustworthiness and rigor. Snowball sampling will also be used, where there is a need to locate specific midwife populations to ensure representation from the various career stages, as well as sufficient participant numbers. Theoretical sampling will be performed to verify and test the evolving study, and will involve inviting new participants to the study to inform this process.

**Figure 1:**
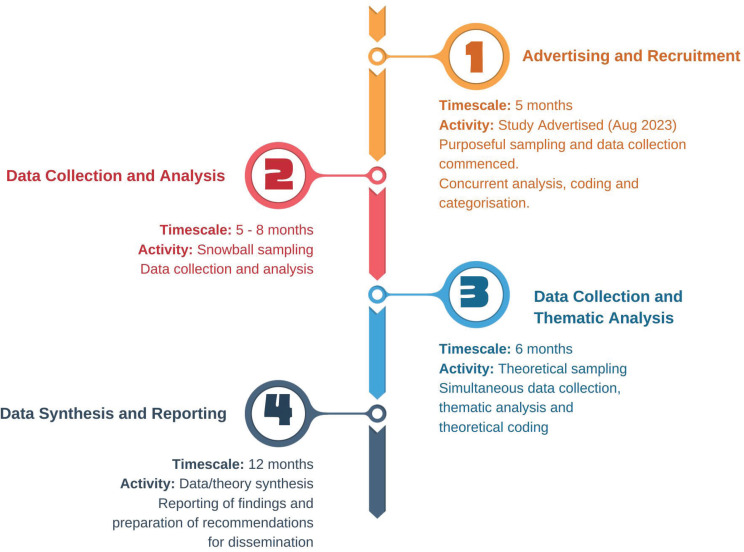
Study timeline

Participants of the study must be currently registered and practicing as a midwife. The sample will not exclude any practice settings or midwives who work in any particular model of care or practice environment.

### Data collection

Basic demographic data will be collected at the point of recruitment to the study. Participant demographic information will include the year of birth, gender, years of midwifery registration, place and area of practice, model of care, and hours worked per week. These data will provide context and allow for stratification and grouping of the study participants. Consistent with GT methods, semi-structured, in-depth interviews will be used to gather the qualitative data set. Open-ended questions will provide a gentle semi-structured framework for the interviews; however, the interviewer will be responsive to the emerging conversation and contributions of participants and will follow their cues to pursue data in response to the research objectives. General topics of discussion will center around the perceived significance of professional relationships and connections in relation to participants’ midwifery practice, psychological well-being, job satisfaction, and intention to remain in or leave the profession. Interviews will either be conducted face-to-face or via video conferencing technology in order to offer flexibility around time availability and promote inclusiveness for rural/interstate participants. Interviews will be audio recorded with participants’ written informed consent. Detailed notes, reflections, and minutes will also be recorded by the researcher to capture emerging concepts that may inform theory development^28^.

Extensive steps will be taken to ensure participant confidentiality is maintained across all phases of the study, including participant registration, data collection, data analysis, reporting, and the safe storage and disposal of all research data in keeping with university policy, standards, and requirements. All participant demographic information and interviews will be de-identified, with each participant receiving a participant reference number to ensure participant anonymity and preservation of confidentiality. While participant interviews will provide the first data set, observations, policies, artifacts, blogs, memos, videos, literature, documents, and informal conversations may also contribute to the ‘all is data’ mandate^29^, where any form of data may be utilized to illuminate the phenomenon of interest.

### Data analysis

Data will be systematically collected and simultaneously analyzed using constant comparative analysis^30^. The coding of data is a fundamental part of the GT data analysis process and is central to developing an emergent theory^31^. Analysis of the data will involve three levels of coding consistent with the chosen methodological approach. These include open coding, selective coding, and theoretical coding, with the aim that this will lead to the discovery of the basic social process employed by participants and the development of the grounded theory that explains the phenomenon of interest^29^. It is essential that this process is seen and utilized as a continuously integrated cycle of data collection, analysis, and sampling^32^. In addition, the researcher will integrate and apply a social-ecological framework in the analysis of data, adapting the socio-ecological spheres of influence (micro, meso, and macro spheres of influence) as a coding family^33^. Such an approach is both sympathetic and compatible with GT processes.

Open coding is a process of coding that discovers themes and concepts from data. Interview data will be transcribed verbatim and meticulously analyzed line-by-line in order to inductively identify themes, patterns, concepts, and reoccurrence^30^. The process involves breaking down, comparing, examining, categorizing, and conceptualizing data^34^. The analysis of each line, sentence, or incident, provides a source of data to be coded into ‘as many categories as possible’^29^. From this rigorous analysis process, conceptual labels that form theoretical codes are created. Simultaneously, the candidate will discuss the analysis with supervisors to ensure and strengthen the analysis process. Participant feedback will be sought to verify that the categories are representative. A process borrowed from phenomenology, member checking will be undertaken as a means to validate data and reduce the potential for research bias. Interview transcripts and/or analyzed data will be returned to participants for verification, ensuring a robust process to further authenticate the emergent theory as representative and trustworthy^35^.

In the Selective Coding stage of the coding process, a core category is created. A core category is a central area of focus around which all other categories integrate and intersect^34^. In addition, selective coding enables the researcher to identify categories that require further refinement, development, and additional data collection, which can occur through theoretical sampling. Theoretical sampling allows the researcher to pursue leads in the data through further collection of data or refinement of interview questions to reduce extraneous data^30,36^. This process should continue until no new categories can be identified, a state known as theoretical saturation.

Theoretical coding is viewed as the final stage of achieving a GT. Theoretical coding integrates and distills the categories that arrive from the substantive coding and analysis phases to support the emergence of a theory. Birks and Mills^36^ note that the researcher who seeks a grounded theory becomes so in tune with the data that they develop a ‘theoretical sensitivity’ to recognize pertinent data to support the emergent theory. Glaser and Strauss^29^ state that the researcher must trust in the emergence of the theory in order for it to be discovered from the data.

The process of memoing is employed to support the analytical process. Memos, which are the researcher’s reflective notes, provide early prompts in the coding process to analyze and explore their properties and relativity, and code data into categories^32^. They form an architectural foundation of documented ideas that serve to provide an insight and auditable trail of the researcher’s thoughts, insights, feelings, and contemplations^30^. Glaser and Strauss^29^ wrote that memos provide an ‘immediate illustration of an idea’. In this study, memos will be used to highlight early themes and to capture ideas and nuances requiring further exploration.

### Rigour in analysis

This study will be guided by an experienced Grounded Theorist to ensure adherence to the methodology. Trustworthiness will be achieved and maintained through the adoption of five criteria, which comprise the gold quality standards of qualitative research. These include credibility, transferability, dependability, conformability, and authenticity^28,34^.

*Credibility*

This will be achieved through the process of member checking respondent validation and in alignment with research and data integrity protocols, to ensure all research participants are described and represented accurately^28^. As the data are being derived from the study of a small and nuanced group, transferability will be achieved by providing in-depth and rich descriptions and through the use of purposive sampling and data saturation^34^.

*Dependability*

This aims to achieve data that maintain stability over time and may apply to different conditions^28^. This is achieved with triangulation, the illumination of the phenomenon from as many different angles and perspectives as possible^28^.

*Conformability*

This is achieved when data are interpreted objectively, with the aim of minimizing the influence of the inquirer.

*Authenticity*

This is supported by conformability and vice versa. Authenticity is achieved with the faithful representation of participant voices as opposed to the researcher voice^21^.

At each point, the lead researcher will strive to be reflexive. This will be achieved by acknowledging and scrutinizing their role in the research process to avoid influence on decisions and interpretations of data^31^. The researcher will use the bracketing approach, borrowed from the Phenomenology methodology, to ensure that, to the best of their ability, personal judgments and assumptions are suspended to allow focus on the analysis and interpretation of data^36^. In addition, the co-authors are experienced qualitative analysts and well-practiced at managing personal biases.

### Ethics and dissemination

Ethics approval was received from the Australian Catholic University Human Research Ethics Committee (ACU HREC) on 20 June 2023 (Ethics Registration Number: 2023-3194E). The study will be conducted with strict and diligent adherence to all relevant Human Research Ethical principles. All participants will be assured of confidentiality and anonymity as outlined in the participant information sheet and all affiliated study information. Participants will also be informed that participation in the study is voluntary, and they can withdraw at any stage of the research process with no penalty or consequence. Each participant will provide written informed consent prior to participating in the interview and study process. The lead researcher (LM) will collect all data, seek to create a safe and private space to conduct the interviews, and endeavor to establish a connection with each participant to promote feelings of trust and comfort, to support honest and relaxed conversation. Although not intended, the interview questions may provoke feelings of anxiety or distress for some participants, and measures will be taken to ensure that appropriate support/referrals are offered if required. In addition, participants will be made aware that they can pause/stop the conversation at any time should they begin to feel unsafe, anxious, or distressed.

Research findings will be shared via the following modes of dissemination: online, workshops, student and professional forums, conferences (local, national, and international), and peer-reviewed publications and reports. The findings may also contribute to further research in affiliated areas of inquiry.

## DISCUSSION

The generation of a theory in relation to the phenomenon being investigated will contribute to an understanding of the impact of professional connection and midwifery relationships, which may influence the well-being and resilience of midwives as well as workforce retention and attrition. The inclusion of a Stakeholder Consultant will help to ensure the validity and application of the findings as they relate and translate to the midwifery profession and practice. Potential limitations and challenges may also arise around the recruitment of sufficient participant numbers and variation. The sturdy methodology provides for theoretical and snowball sampling to help mitigate this challenge.

### Study status

Recruitment of study participants commenced in August 2023.

## CONCLUSIONS

It is anticipated that the research findings will have national and international implications to help identify the impacts, including benefits and any potential disadvantages, of professional relationships in sustaining midwifery careers. This is for the purpose of understanding how to better support midwives and improve their well-being and retention in the workforce. In addition, the findings will strengthen knowledge of the phenomenon, and contribute to and help inform further change in this field of inquiry.

## Data Availability

Raw data will not be made available in the interests of protecting the privacy and confidentiality of study participants. This is in alignment with the approval given by the Human Research Ethics Committee. De-identified and synthesized data will form the basis of the developing theory and will, therefore, be available through the dissemination of study findings.
