# Erratum: How do professional connections and relationships impact midwives’ well-being and career sustainability? A Grounded Theory study protocol

**DOI:** 10.18332/ejm/186079

**Published:** 2024-03-28

**Authors:** European Publishing Production Team

**Affiliations:** 1 European Publishing Production Team, Heraklion, Greece

**Keywords:** midwifery, midwives, well-being, career sustainability, relationships, professional connections

**Erratum on:**

**How do professional connections and relationships impact midwives’ well-being and career sustainability? A Grounded Theory study protocol**

**By Lynnelle Moran, Sara Bayes, Kim Foster**

**European Journal of Midwifery, Volume 8, Issue March, Pages 1-7**

**Publish date: 4 March 2024**

DOI: https://doi.org/10.18332/ejm/178385

In the original article, on the fifth page, in the **Ethics and dissemination section**, the Ethics Committee and the registration number were missing as shown below:

Ethics approval was received from the [redacted for review] Human Research Ethics Committee on June 20, 2023 (Ethics Registration Number [Redacted for review]).

They have been now corrected as follows:

Ethics approval was received from the Australian Catholic University Human Research Ethics Committee (ACU HREC) on 20 June 2023 (Ethics Registration Number: 2023-3194E).

The mentioned changes are corrected also online.

